# Cervical HPV type-specific pre-vaccination prevalence and age distribution in Croatia

**DOI:** 10.1371/journal.pone.0180480

**Published:** 2017-07-10

**Authors:** Ivan Sabol, Nina Milutin Gašperov, Mihaela Matovina, Ksenija Božinović, Goran Grubišić, Ivan Fistonić, Dragan Belci, Laia Alemany, Sonja Džebro, Mara Dominis, Mario Šekerija, Sara Tous, Silvia de Sanjosé, Magdalena Grce

**Affiliations:** 1 Department of Molecular Medicine, Ruđer Bošković Institute, Zagreb, Croatia; 2 University Hospital Sisters of Mercy, Clinic of Obstetrics and Gynaecology, Zagreb, Croatia; 3 Obstetrics, Gynecology and Menopause Clinic, Zagreb, Croatia; 4 Department of Gynecology and Obstetrics, General Hospital Pula, Pula, Croatia; 5 Cancer Epidemiology Research Program, Unit of Infections and Cancer, Catalan Institute of Oncology, Barcelona, Spain; 6 CIBER en Epidemiología y Salud Pública (CIBERESP), Barcelona, Spain; 7 Department of Pathology and Cytology, University of Zagreb, School of Medicine, University Hospital Merkur, Zagreb, Croatia; 8 Croatian National Cancer Registry, Croatian Institute of Public Health, Zagreb, Croatia; 9 School of Medicine, Andrija Štampar School of Public Health, University of Zagreb, Zagreb, Croatia; Fondazione IRCCS Istituto Nazionale dei Tumori, ITALY

## Abstract

The main etiological factor of precancerous lesion and invasive cervical cancer are oncogenic human papillomaviruses types (HPVs). The objective of this study was to establish the distribution of the most common HPVs in different cervical lesions and cancer prior to the implementation of organized population-based cervical screening and HPV vaccination in Croatia. In this study, 4,432 cervical specimens, collected through a 16-year period, were tested for the presence of HPV-DNA by polymerase chain reaction (PCR) with three sets of broad-spectrum primers and type-specific primers for most common low-risk (LR) types (HPV-6, 11) and the most common high-risk (HR) types (HPV-16, 18, 31, 33, 45, 52, 58). Additional 35 archival formalin-fixed, paraffin embedded tissue of cervical cancer specimens were analyzed using LiPA_25_ assay. The highest age-specific HPV-prevalence was in the group 18–24 years, which decreased continuously with age (*P*<0.0001) regardless of the cytological diagnosis. The prevalence of HR-HPV types significantly increased (*P*<0.0001) with the severity of cervical lesions. HPV-16 was the most common type found with a prevalence (with or without another HPV-type) of 6.9% in normal cytology, 15.5% in atypical squamous cells of undetermined significance, 14.4% in low-grade squamous intraepithelial lesions, 33.3% in high-grade squamous intraepithelial lesions, and 60.9% in cervical cancer specimens (*P*<0.0001). This study provides comprehensive and extensive data on the distribution of the most common HPV types among Croatian women, which will enable to predict and to monitor the impact of HPV-vaccination and to design effective screening strategies in Croatia.

## Introduction

Cervical cancer represents an important public health issue in Croatia where it is the ninth most common type of cancer in women, and also ninth most common cause of cancer death [1]. Each year in Croatia, over 300 women develop cervical cancer and approximately 130 women die from this disease. According to the latest data for Croatia, in 2014 there were 307 new cases (world age-standardized incidence rate (ASR-W) 11.9/100,000 women-years/WY), and in the same year 130 women died from cervical cancer (ASR-W 4.4/100,000 WY). In Europe, cervical cancer is estimated to be the sixth most common cancer in women in 2012, with almost 60,000 new cases per year (3.6% of all incident cancers; 11.4 ASR-W) [2,3].

**Fig 1:**
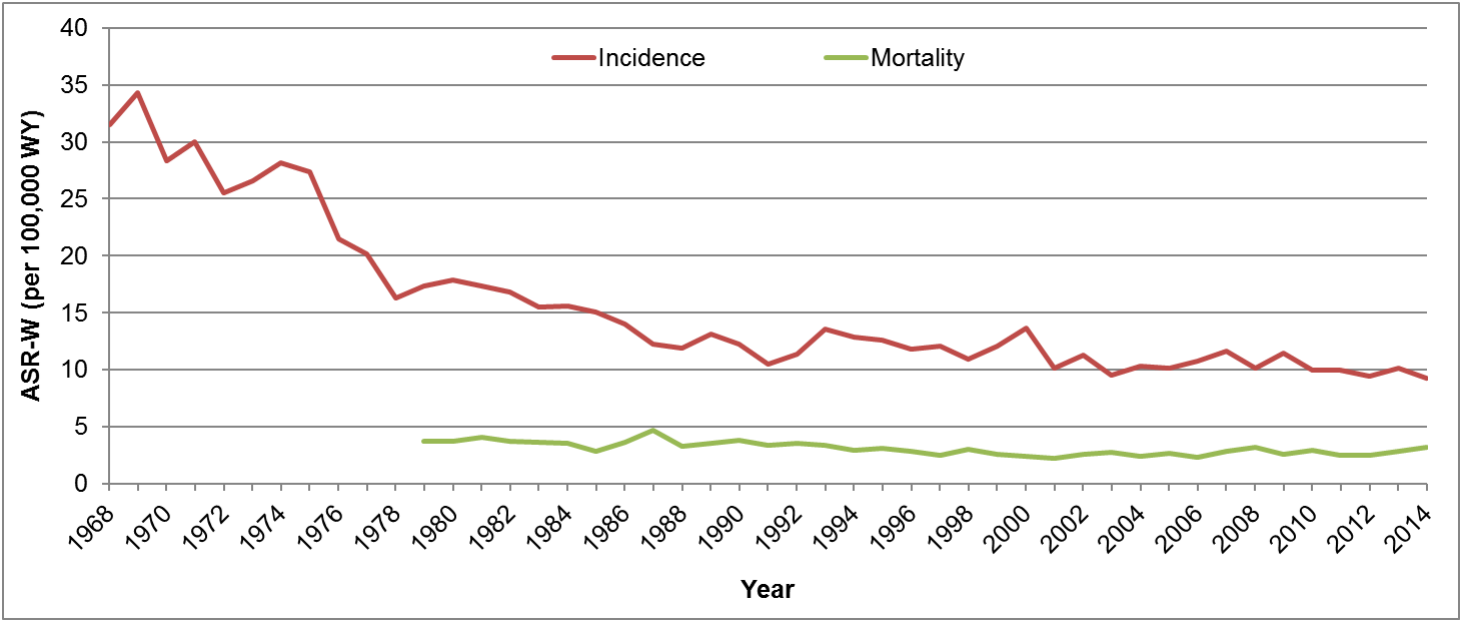
World age-standardized incidence and mortality rates (per 100,000 women-years) of cervical cancer in Croatia from 1968 to 2014 [1].

Since the introduction of the opportunistic cervical cancer screening in Croatia trends of cervical cancer rates were declining as shown from 1968 to 2014 on Fig 1 [1]. The trends in the cervical cancer mortality rates in Croatia remained at a low level but no decrease was observed over the last two decades (Fig 1). Age-specific incidence rates of cervical cancer in Croatia was unchanged from 1988 to 2013,showing two distinctive peaks with the highest rate at age 50 and 75 [1]. Moreover, the results from EUROCARE-5 [4], a study on cancer 5-year relative survival in Europe, showed that Croatia is a little bit above average (65.1% *vs*. 62.4%) when compared to other European countries.

Based on the unsatisfying situation in Croatia regarding cervical cancer, efforts were made to improve cervical cancer prevention in general. Thus, an organized nation-wide screening program for cervical cancer was implemented in 2012 under the supervision of the Croatian National Institute of Public Health (www.hzjz.hr) where the target group are women age 20 to 64 being screened by the conventional Pap smear every third year [5,6]. In addition, the HPV-vaccines have become available and recommended since 2007 [6,7], and an organized nation-wide HPV-vaccination program was introduced in 2016, also under the supervision of the Croatian National Institute of Public Health.

Human papillomavirus (HPV) is now understood to be necessary, but insufficient for the development of cervical cancer [8]. Nowadays, within the family *Papillomaviridae*, more than 200 HPV-types are well characterized [9]. Over 40 types (*Alphapapillomavirus* genus) infect the female anogenital region, of which some cause benign genital warts and others may lead to precursor cervical lesions, cervical intraepithelial neoplasia (CIN) and cervical cancer [10]. Of these, at least 12 are significantly associated with progression of CIN to cervical cancer and are considered carcinogenic to humans. Thus, HPV-types 16, 18, 31, 33, 35, 39, 45, 51, 52, 56, 58, and 59 are classified as carcinogenic to humans (Group 1; usually named high-risk, HR), HPV-68 as probably carcinogenic to humans (Group 2A), HPV-types 26, 30, 34, 53, 66, 67, 69, 70, 73, 82, 85 and 97 (Group 2B) as possibly carcinogenic to humans, while HPV-6 and 11 (Group 3; usually named low-risk, LR) are not classifiable as to their carcinogenicity to humans [11]. Most of HR-HPV-types are phylogenetically related to either HPV-16 (carcinogenic types 31, 33, 35, 52 and 58) or HPV-18 (carcinogenic types 39, 45 and 59, and probably carcinogenic 68) [12].

It is essential for each country to evaluate the prevalence of HPV-types before large scale implementation of prophylactic HPV-vaccines, to perform HPV-testing in clinical practice and screening, and to monitor the impact on cervical cancer control in the population [13–15]. These data will be useful to prospectively estimate the effectiveness of HPV-vaccination, and assess changes in the incidence and distribution of HPV-types. In addition, as HPV-infections will decline in the vaccinated population, HPV-testing as primary test for cervical screening will probably be a better tool to identify women at risk of developing cervical cancer [16].

This paper describes the results of a survey of fresh cervical specimens, collected between 1999 to 2015 in Croatia, which were tested using consensus and type-specific amplification to estimate the prevalence (the proportion of infected specimens) and the age distribution of the most common HPV-types, i.e. the LR-HPV-6 and 11, the HR-HPV-16, 18, 31, 33, 45, 52, and 58. In addition, the formalin-fixed, paraffin embedded (FFPE) cervical cancer specimens from women diagnosed with cervical cancer between 1982 and 1995 were included in this study.

## Material and methods

### Study group

The fresh cervical cytobrush specimens were collected for HPV detection and typing in different gynecological clinics in Zagreb (Clinic of Obstetrics and Gynecology of the University Hospital Sisters of Mercy; Obstetrics, Gynecology and Menopause Clinic) and Pula (Department of Gynecology and Obstetrics of the General Hospital Pula), Croatia, while FFPE cervical cancer tissue were collected at the Department of Pathology and Cytology, University Hospital Merkur, Zagreb, Croatia. In the 16-year period, from 1999 to 2015, 4,562 women were referred for HPV testing at the Ruđer Bošković Institute of which 4,432 DNA samples were successfully analyzed. In most cases (82.9%) the cytological diagnosis was available [17]. Women who attended those clinics came from all over the country although living in the city of Zagreb and surroundings, where more than a quarter of the Croatian population lives, and it represents a mixture of rural (28%) and urban population (72%) [18]. There were 11 cervical scrapes from women referred for treatment of cervical cancer taken immediately before the procedure. An additional 35 FFPE tissue of cervical cancer specimens from women diagnosed from 1982 to 1995 were histopathologically evaluated as previously described [19]. Thus, a total of 4,467 cervical samples were available for HPV analysis and typing.

Part of cervical DNA specimens collected for research purpose were obtained from the Croatian Tumor and DNA Bank for basic research, Ruđer Boković Institute, Zagreb, Croatia [20]. Part of fresh cervical samples for HPV diagnostics and research were collected at the Sisters of Mercy Hospital and received the official institutional and ethical approval (Klinička bolnica “Sestre milosrdnice”, PRO-31-06). The archival FFPE samples were collected at the University Hospital Merkur specifically for the international study RIS HPV TT coordinated at ICO (Institut Català d’Oncologia, Barcelona, Spain) [19]. Written patient/participant consent was not necessary because each cervical sample is accompanied by the Laboratory service request forms, which have to be signed and approved by the practicing physician responsible for the verbal patient/participant consent, which was obtained for each cervical specimen that was collected both for HPV diagnostic and research purposes, and the Bioethical Board of Ruđer Bošković Institute (BP-021-227/2-2005) approved it. The handling and publication of patients’ data in this study were strictly in accordance with the Declaration of Helsinki DoH/Oct2008 including confidentiality and anonymity.

### DNA preparation

DNA from cervical cell samples was isolated in two ways: by standard phenol-chloroform extraction previously described [21,22] until 2006, and further on by purification on BioRobot EZ1 according to the manufacturer’s instruction (Qiagen, Hilden, Germany). After DNA extraction, the purified DNA was dissolved in 50–100 μl of tridistillated sterile water and stored at –20°C until further analysis. Each DNA was analysed by electrophoresis on 1% agarose gels and/or spectrophotometrically [23]. DNA from FFPE tissue was processed as previously described [19].

### HPV detection and typing

For cervical cell samples previously established method was used [24]. Briefly, three sets of the consensus primers were used: PGMY09/MY11, L1C1/L1C2-1/L1C2-2 and GP5/6 consensus primers. The quality of the isolated DNA was tested by amplification of the 268 bp sequence of β-globin gene using PC04/GH20 primers [25] in a multiplex PCR with PGMY primers. Literature derived, type-specific (TS) primers for HPV-6/11, 16, 18, 31, 33, 45, 52 and 58 were also used in three separate multiplex PCR reactions (HPV-6/11 and 31; HPV-16, 18 and 33; HPV-52 and 58) and one single PCR to amplify HPV-45 [24]. The reaction mixture contained tri-distillated sterile water, 10 mM Tris–HCl (pH 8.3), 50 mM KCl, 1.5 mM MgCl_2_, 100 μM of each dNTP, 0.15 μM of each TS primer, 0.12 U AmpliTaq Gold DNA Polymerase (Roche) and 100 ng of each DNA in a total volume of 20 μl. Each PCR was carried out with first denaturation step at 95°C for 10 min and final extension at 72°C for 15 min. The conditions and the number of denaturation-annealing-extension cycles for each set of primers were previously described [24]. Aliquots of each PCR product (10 μl) were analysed by electrophoresis on 2% agarose gels stained with Ethidium Bromide. The amplified products were visualized by UV irradiation of the gels and photographed by Image Master VDS (Pharmacia Biotech). Any sample positive for the consensus PCR but negative for all type specific reactions was classified as undetermined type, HPV-X.

HPV-DNA detection and genotyping in FFPE tissue was performed as previously described [19]. Briefly, SPF-10 broad-spectrum primers directed PCR followed by DNA immunoassay in the reverse hybridisation line probe assay (LiPA_25_; Laboratory Biomedical Products, Rijswijk, Netherlands) were used according to the manufacturer’s recommendations. The LiPA_25_ assay was used for genotyping allowing the identification of 25 HR and LR-HPV-types (6, 11, 16, 18, 31, 33, 34, 35, 39, 40, 42, 43, 44, 45, 51, 52, 53, 54, 56, 58, 59, 66, 68, 70, and 74).

### Statistical analysis

The standard Chi-square (χ2) test was used to study associations between two variables. Two-tailed *P* values were calculated in 2 x 2 tables using the GraphPad Prism Software (version 4.00; San Diego, California, USA [http://www.graphpad.com]). Tests for trend were done using Chi-square (χ2) test for trend. All tests were two sided and the significance level was set at *P*<0.05.

## Results

The mean age of the study population was 35.5 years (range from 18 to 85 years); 6% of samples with unrecorded age. Among 4,198 women with known age, 72.8% belonged to the recommended target age group for screening, 25 to 64 years [15]. In addition, there were 26.2% younger women (18–24 years) and almost 1% women 65 years and older.

For 17.1% of 4,467 processed cervical samples the cytological diagnosis was unknown (Table 1). The rest of the study group consisted of 1.6% normal cytology, 23% ASCUS (atypical squamous cells of undetermined significance), 29.3% LSIL (low-grade squamous intraepithelial lesion), 27.9% HSIL (high-grade squamous intraepithelial lesion), and 1% cervical cancer samples.

**Table 1:** Distribution of HPV-infection, type-specific prevalence and age range by cytological/histological diagnosis (N = 4,467).

		Unknown diagnosis		Cytological diagnosis^c^								Histopathological diagnosis		Total	
				Normal		ASCUS		LSIL		HSIL		Cervical cancer			
		N	%	N	%	N	%	N	%	N	%	N	%	N	%
Cases		764	(17.1%)	72	(1.6%)	1029	(23%)	1310	(29.3%)	1246	(27.9%)	46	(1%)	4467	(100%)
Distribution of HPV-infections	Any HPV	353	(46.2%)	15	(20.8%)	517	(50.2%)	745	(56.9%)	980	(78.7%)	42	(91.3%)	2652	(59.4%)
	Untyped-HPV^d^	104	(13.6%)	6	(8.3%)	156	(15.2%)	285	(21.8%)	243	(19.5%)	2	(4.3%)	796	(17.8%)
	HR-HPV^a^	168	(22%)	8	(11.1%)	322	(31.3%)	390	(29.8%)	692	(55.5%)	40	(87%)	1620	(36.3%)
	Single HPV	205	(26.8%)	8	(11.1%)	269	(26.1%)	361	(27.6%)	530	(42.5%)	36	(78.3%)	1409	(31.5%)
	Multiple HPVs	44	(5.8%)	1	(1.4%)	92	(8.9%)	99	(7.6%)	207	(16.6%)	4	(8.7%)	447	(10%)
HPV-type specific prevalence	HPV-6/11^b^	103	(13.5%)	1	(1.4%)	72	(7%)	111	(8.5%)	101	(8.1%)	0	(0%)	388	(8.7%)
	HPV-16	88	(11.5%)	5	(6.9%)	159	(15.5%)	188	(14.4%)	415	(33.3%)	28	(60.9%)	883	(19.8%)
	HPV-18	16	(2.1%)	0	(0%)	32	(3.1%)	47	(3.6%)	71	(5.7%)	4	(8.7%)	170	(3.8%)
	HPV-31	31	(4.1%)	1	(1.4%)	90	(8.7%)	83	(6.3%)	166	(13.3%)	1	(2.2%)	372	(8.3%)
	HPV-33	11	(1.4%)	0	(0%)	41	(4%)	29	(2.2%)	54	(4.3%)	5	(10.9%)	140	(3.1%)
	HPV-45	12	(1.6%)	1	(1.4%)	14	(1.4%)	27	(2.1%)	38	(3%)	5	(10.9%)	97	(2.2%)
	HPV-52	26	(3.4%)	2	(2.8%)	41	(4%)	61	(4.7%)	85	(6.8%)	0	(0%)	215	(4.8%)
	HPV-58	16	(2.1%)	0	(0%)	30	(2.9%)	40	(3.1%)	60	(4.8%)	1	(2.2%)	147	(3.3%)
Average age (range)		34	(18–79)	32	(18–70)	33	(18–76)	31	(18–75)	31	(18–71)	50	(31–85)	35	(18–85)

^a^ HR (high-risk) HPV-types 16, 18, 31, 33, 45, 52 and 58.

^b^ LR (low-risk) HPV-types 6 or 11.

^c^ ASCUS atypical squamous cells of unknown significance, LSIL low grade squamous intraepithelial lesion, HSIL high grade squamous intraepithelial lesion.

^d^ one cervical cancer sample was typed as HPV-68 or 73 according to LiPA_25_ assay (version 1).

DNA from fresh samples was suitable for HPV-DNA analysis (positive β-globin amplification). Out of 4,467 samples, 2,652 (59.4%) were positive for HPV-DNA, of which 1,621 (36.3%) were positive for at least one HR-HPV-type (HPV-16, 18, 31, 33, 45, 52 and 58), while 388 (8.7%) were positive to LR-HPV-6 or 11 (Table 1). In addition, there were 796 (17.8%) samples in which HPV-infections were detected by consensus PCR reactions but the types remained undetermined (HPV-X) (Table 1).

Single infection prevalence was 31.5% (1,409/4,467), while multiple infection prevalence was 10% (447 of 4,467) (Table 2). The most frequent genotype in single and multiple infections was HPV-16, found in 13.3% and 6.4% cases, respectively. The type-specific prevalence of HPV-16 in the study population was 19.8%, followed by HPV-6/11 (8.7%), 31 (8.3%), 52 (4.8%), 18 (3.8%), 58 (3.3%), 33 (3.1%) and 45 (2.2%) (Table 2).

**Table 2:** Distribution of specific HPV-types in single and multiple infections among all samples of the study population (N = 4,467).

	No. Samples (%)	HR-HPV^a^	LR-HPV^b^	HPV-16	HPV-18	HPV-31	HPV-33	HPV-45	HPV-52	HPV-58
Single infections	1409	1173	236	596	80	217	71	43	97	69
	(31.5%)	(26.3%)	(5.3%)	(13.3%)	(1.8%)	(4.9%)	(1.6%)	(1.0%)	(2.2%)	(1.5%)
Multiple infections	447	447	152	287	90	155	69	54	118	78
	(10.0%)	(10.0%)	(3.4%)	(6.4%)	(2.0%)	(3.5%)	(1.5%)	(1.2%)	(2.6%)	(1.7%)
Total prevalence	2652	1620	388	883	170	372	140	97	215	147
	(59.4%)	(36.3%)	(8.7%)	(19.8%)	(3.8%)	(8.3%)	(3.1%)	(2.2%)	(4.8%)	(3.3%)

^a^HR (high-risk) HPV-types, any of 16, 18, 31, 33, 45, 52 and/or 58.

^b^ LR (low-risk) HPV-types 6 and/or 11.

**Fig 2:**
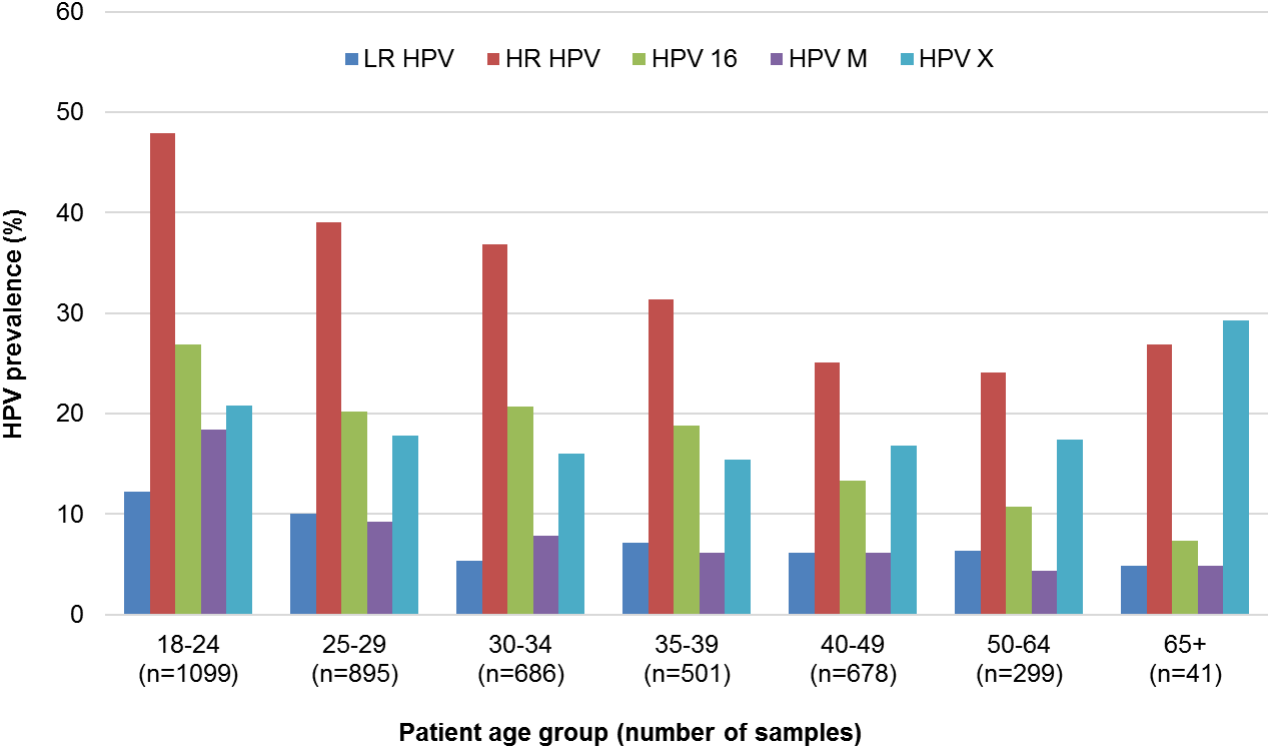
Prevalence of HPV-infection according to patient age.

Majority of multiple infections contained only 2 types (350/447 = 78.3; median number of types 2; Inter Quartile Range (IQR) 2–2), while some samples were positive for maximally 5 different types. The distribution of HPV-infections by age is shown in Fig 2. The prevalence of HR-HPVs, multiple HPVs and LR-HPVs was the highest in the age group 18 to 24 years, while for HPV-X the highest prevalence was in the age group 65+ years. The prevalence of HPV-infection in general, HR-HPVs, LR-HPVs, multiple HPVs is decreasing significantly (*P*<0.0001) with age, while those of HPV-X, less abundant than HR-HPV-types, is not decreasing significantly (*P* = 0.0678) with age and is even slightly increasing after the age of 39 years.

**Fig 3:**
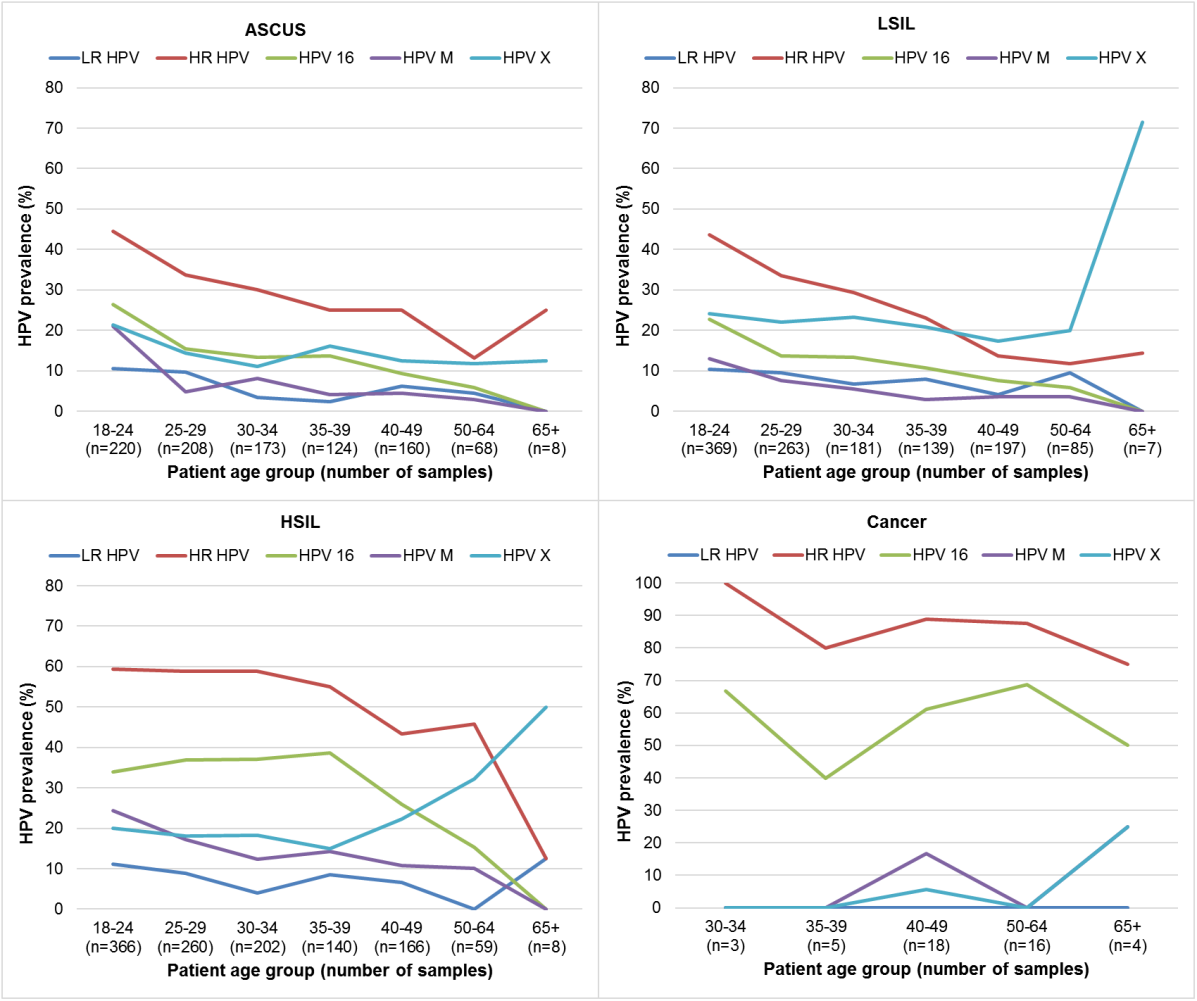
Age-specific prevalence of HPV-infection according to cytological/pathological diagnosis.

The type-specific HPV prevalence according to the diagnosis is shown in Table 1. The HSIL diagnosis is mostly attributed to HPV-16 found in 33.3% cases, and then to HPV-31 (13.3%). They were followed by HPV-6/11 (8.1%), HPV-52 (6.8%), HPV-18 (5.7%), HPV-58 (4.8%), HPV-33 (4.3%) and HPV-45 (3.0%). HPV-16 was also the most frequently found type in LSIL and ASCUS diagnosis, identified in 14.4 and 15.5% cases, respectively. Afterward, HPV-6/11, 31, 52, 18, 58, 33 and 45 were found in LSIL diagnosis by decreasing prevalence (from 8.5 to 2%), while HPV-31, 6/11, 33, 52, 18, 58, and 45 (prevalence ranging from 9 to 1.3%) in ASCUS diagnosis. The distribution of HPV-infection according to the severity of the cervical diagnosis shows a significant (*P*<0.0001) increase of HPV-infection in general, multiple HPVs and HR-HPVs, notably HPV-16 and HPV-31 (Table 1). The increasing trend of HPV-X (*P* = 0.014) was also significant (Table 1). The age-specific distribution of HPV-infection according to the severity of the cytological diagnosis is shown on Fig 3.

## Discussion

This study provides extensive and comprehensive information about the distribution of the most prevalent HPV-types among Croatian women, which are in line with our previous small scale studies [21,22]. Such a study is crucial prior to introduce organized HPV-vaccination as primary, and organized screening as secondary cervical cancer prevention programs [15].

There were 59.4% HPV-positive cervical DNA samples, most of which could be correlated to age and cervical diagnosis of the tested women. Taking into account the multiple HPVs (10%), the prevalence of HR-HPVs was 36.3%, while those of LR-HPVs was only 8.7%. The most frequent type in each age group and diagnosis including normal cytology and cancer was HPV-16, found in 19.8% of samples. Similar findings were reported by other recent studies; summary of 18 studies from 14 European countries (mostly northern and western Europe) indicated that HPV-16 is the most prevalent HPV-type, found in 29.8% of HPV-positive samples (range 19–43%) [26]. The world-wide meta-analysis of distribution of HPV-types, also points out HPV-16, as the most prevalent type, whose presence is steadily increasing with the severity of the cytological changes, from normal cytology (20.4% +/- 3.6%), to ASCUS (22.9% +/- 2.9%), LSIL (25.1% +/- 2.8%), and HSIL (47.5% +/- 5.5%), and being the highest in ICC (62.6 +/- 2.2) [27]. The second most common type in this study was HPV-31 (samples diagnosed ASCUS and HSIL), except in the sub-group of LSIL diagnosis where HPV-6/11 was the second most common type and HPV-31 ranked third. This is in concordance with the results of the large scale study of the HPV prevalence in Netherlands showing HPV-16 as the most prevalent HR-HPV-type, followed by HPV-31 [28]. Similarly, the study of Arbyn et al. [29] on a large collection of samples from Belgium indicated HPV-16 in 32% of HSIL cases, followed by HPV-31 found in 22% of cases. However, the percentage of HPV-31 in their study was considerably higher than this one. Finally, the study of Guan et al. [27], a world-wide meta-analysis support our finding of HPV-31 to be the second most common type with the positivity slightly increasing from normal (8.0+/-2.0) to HSIL (11.0 +/- 1.6), but significantly lower in ICC (4.0 +/- 0.4).

It is interesting to note that HPV-52, phylogenetically close to HPV-31 (*Alphapapillomavirus* genus, *HPV-16* species) [30] was the fourth most common HPV-type found in HSIL and LSIL samples and the fifth most common HPV-type in ASCUS. Similarly, HPV-58, phylogenetically close to HPV-33 (*Alphapapillomavirus* genus, *HPV-16* species) [30] was the sixth most common in the sub-groups HSIL and LSIL and the seventh most common type in the sub-group of ASCUS. Because of their relative high prevalence, HPV-52 and 58 need special attention regarding the eventual cross-protection by HPV-vaccines as proven for HPV-31, 33 and 45 [31].

Contrary to the expectations [32], HPV-18 and 45 (*Alphapapillomavirus* genus, *HPV-18* species) [30] were the less common HR types in HSIL in cervical samples of Croatian women, ranking fifth and eighth, respectively. Other studies in European countries [29,33,34] found these HPV-types less frequently in HSIL than in cervical cancer. These two HR-HPV-types are frequently found in adenocarcinomas of the cervix, which represents 1/5 of cervical cancer cases in Europe [35].

The age distribution of HPV-infections is similar to those reported in most studies, high among young women and gradually decreasing with age. This trend was slightly different for undetermined HPV-types that are presumably also the less common types; i. e. their age dependent decrease is reversed after the age of 39 years. This finding is in line with other European studies where the overall HPV prevalence shows two age related peaks, one in the twenties and the other in the forties [29,36]. The question remains, which types (low or high-risk) are included in this category of undefined HPV-types and how abundant are they among older women. It is important to note that this second peak of higher prevalence of HPV was also observed with HR-HPV-types after the age of 59, while multiple infection and LR-HPV-types are linked to younger age [37]. Therefore, the second peak of undetermined-HPV might also correspond to HR-HPV-types that generally tend to persist in the older women. Indeed, in the previous studies, the less common types among Croatian women with HSIL were probable HR types 53 and 66, and HR-HPV-58, 56 and 52, followed by other, even rarer, types [38]. These findings are not irrelevant since [39] detected some cases of cervical abnormalities in women age 50+ and with former negative smears. Therefore, not only HPV detection but also HPV typing of a broader spectrum of types [40] should be considered in the diagnostic algorithm of women ≥50 years to determine the attributed risk by a particular HPV-type.

Similar distribution of HR-HPV-types according to patient age and cytological diagnosis is shown in other studies [10,28,29]. This trend was significant for HPV-infection in general, HR-HPVs, multiple HPVs, undetermined-HPVs and specific HR-HPVs, particularly HPV-16 and HPV-31, except for HPV-33. The decreasing trend of LR-HPV with the severity of the cytological diagnosis was not significant.

In this study, the majority of multiple HPVs were found among women age ≤25 years (Fig 3), similarly like in other populations [41,42]. However, contrary to those studies, multiple HPVs among Croatian women were surprisingly more linked to high-grade lesions. In addition, Hadzisejdic et al. [43] also found a high prevalence of multiple HPVs in squamous cell carcinoma in comparison to HSIL among Croatian women. This indicates that young women with multiple infections have increased risk of having precancerous cervical lesions and that special attention should be given to them. Here again, HPV typing of a broader spectrum of HPVs has a justified application in the diagnostic algorithm.

In this study HPV-16 was found in 33.3% and 60.9% in HSIL and cancer samples, while HPV-18 was found in 5.7% and 8.7% samples, respectively (Table 1). Therefore, the implementation of prophylactic vaccine with high coverage could lower the number of HSIL lesions by up to 40% and cancer by up to 70%. If we consider that HPV-vaccines show cross reactivity with non-vaccine HPV-types, notably HPV-31, 33 and 45, this could add extra prevention against HSIL and subsequent cancer [31]. Other studies give similar optimistic predictions of the benefit of HPV-vaccination on the overall number of cervical abnormalities [28,44]. However, the proportion of LSIL would not be substantially reduced by vaccination, since HPV-16 and 18 are found in 14.4% and 3.6% of LSIL, and 15.5% and 3.1% of ASCUS, respectively. Sargent et al. [45] also argue that the vaccination is not going to prevent a large number of LSIL, that contain HPV-16 in a much lower percentage, so its overall impact on reduction of cervical abnormalities is going to be much smaller. Also, according to their data, a relatively large proportion of HPV-16 and 18 positive samples contained other HPV types as well (43% of HPV-16 or 18 positive LSIL, and 34% of HSIL were multiple infections), and it is not clear yet to what extent the vaccination would have an impact on these cases [45]. As all HPV-types analyzed in this study are also included in the newest 9-valent HPV-vaccine [46], it can be expected that this vaccine with high enough coverage could prevent up to 70% ASCUS, 62% LSIL, 75% HSIL and 95% cancer cases, as there were that many positive lesions (excluding undetermined-HPVs) in our study.

The strength of this study is the high number of abnormal cytological smears (3,585), most of which (71%) corresponding to the target age group for cervical screening (25 to 64 years). However, the weakness of the study is the very low number of normal cytological smears (N = 72) and limited number of histopathologically confirmed cervical cancer (N = 35). The reason for that is because there were no organized screening program in Croatia at the time of sample collection, only the opportunistic screening was in place, so mostly women with gynecological problems were referred to HPV-testing in this study [47]. Through the years, the Croatian diagnostic and therapeutic algorithm for diagnosis and management of cervical lesions [48], that is in line with international guidelines, has been gradually adopted. Nowadays, HPV-DNA testing in Croatia is mainly used for triage of borderline cytological results and for follow-up after treatment of high-grade cervical lesions.

In conclusion, the study gives an order of importance of the most common high-risk types (HPV-16, 18, 31, 33, 45, 52 and 58) in the Croatian population, which were previously found to be the most common cancer causing types worldwide [35]. The study provides comprehensive and extensive data on the distribution of those HPV-types among Croatian women over a ten-year period. HPV-16 was the most common HPV-genotype in cervical scrapes of Croatian women. The second most frequent among tested types were LR-HPV-6/11, followed by HR-HPV-31, 52, 18, 58, 33 and 45. The majority of HR-HPVs are associated with high-grade cervical lesions, but also with younger women, who are therefore exposed to the risk of developing cervical cancer early in their lives. This study also supports the implementation of prophylactic HPV-vaccination that should significantly decrease the occurrence of HSIL, and consequently lower the number of lesions subjected to treatment and extensive follow-up.
